# Sophisticated expression responses of ZNT1 and MT in response to changes in the expression of ZIPs

**DOI:** 10.1038/s41598-022-10925-2

**Published:** 2022-05-05

**Authors:** Shino Nagamatsu, Yukina Nishito, Hana Yuasa, Nao Yamamoto, Taiki Komori, Takuya Suzuki, Hiroyuki Yasui, Taiho Kambe

**Affiliations:** 1 Division of Integrated Life Science, Graduate School of Biostudies, Kyoto University, Kyoto, 606-8502 Japan; 2 Department of Analytical & Bioinorganic Chemistry, Division of Analytical and Physical Sciences, Kyoto Pharmaceutical University, Kyoto, 607-8414 Japan; 3 Graduate School of Integrated Sciences for Life, Hiroshima University, Higashi-Hiroshima, 739-8528 Japan

**Keywords:** Metals, Ion transport, Membrane proteins

## Abstract

The zinc homeostatic proteins Zn transporter 1 (ZNT1) and metallothionein (MT) function in dampening increases in cytosolic zinc concentrations. Conversely, the expression of ZNT1 and MT is expected to be suppressed during decreases in cytosolic zinc concentrations. Thus, ZNT1/MT homeostatic responses are considered to be essential for maintaining cellular zinc homeostasis because cellular zinc concentrations are readily altered by changes in the expression of several Zrt-/Irt-like proteins (ZIPs) under both physiological and pathological conditions. However, this notion remains to be tested experimentally. Here, we investigated the aforementioned homeostatic process by analyzing ZNT1 and MT protein expression in response to ZIP expression. Overexpression of cell-surface-localized ZIPs, such as ZIP4 and ZIP5, increased the cellular zinc content, which caused an increase in the expression of cell-surface ZNT1 and cytosolic MT in the absence of zinc supplementation in the culture medium. By contrast, elimination of the overexpressed ZIP4 and ZIP5 resulted in decreased expression of ZNT1 but not MT, which suggests that differential regulation of ZNT1 and MT expression at the protein level underlies the homeostatic responses necessary for zinc metabolism under certain conditions. Moreover, increased expression of apically localized ZIP4 facilitated basolateral ZNT1 expression in polarized cells, which indicates that such a coordinated expression mechanism is crucial for vectorial transcellular transport. Our results provide novel insights into the physiological maintenance of cellular zinc homeostasis in response to alterations in cytosolic zinc concentrations caused by changes in the expression of ZIPs.

## Introduction

Cellular zinc homeostasis is maintained by the cooperative functions of two zinc transporter family proteins, Zrt-/Irt-like protein (ZIP) and Zn transporter (ZNT)^1–3^, as well as by the cytosolic zinc-binding protein metallothionein (MT)^4,5^. Fourteen members of ZIPs, 10 members of ZNTs, and 11 isoforms of MTs are functional in humans^1–5^. Most ZIPs localize on the plasma membrane and mediate zinc uptake into the cytosol, and certain ZIPs localize to intracellular compartments and mediate zinc release from the lumen of these compartments. ZIP4 is expressed in the apical membrane of the enterocytes, whereas ZIP5 is localized to the basolateral membrane of the enterocytes and acinar cells; they transport extracellular zinc into the cells^6–9^. In contrast, ZIP7 and ZIP13 localize to the ER or Golgi apparatus, respectively^10–14^. Therefore, ZIPs function in replenishing cytosolic zinc levels. The increases in cytosolic zinc concentrations are counteracted through ZNT-mediated mobilization of zinc either into the lumen of intracellular compartments or out of the cells; therefore, ZNTs function in removing cytosolic zinc^15,16^. The efflux of zinc from the cells is mediated only by ZNT1, because it is the only ZNT localized to the cell surface^17,18^. Furthermore, cytosolic zinc (free zinc ion) levels are also lowered through zinc sequestration by MT^19^. Accordingly, coordination of these processes is regarded as being essential for maintaining cellular zinc concentrations around a specific homeostatic setpoint in each cell type, which must be accomplished in a spatiotemporally appropriate manner^15,20,21^.

ZIP expression is widely reported to be physiologically and pathologically upregulated or downregulated in response to various transient and chronic stimuli, including cytokines and lipopolysaccharide, or pathophysiological conditions^22–30^. Thus, the cellular zinc content can be readily altered through changes in ZIP expression, and ZIP-mediated increases in cytosolic zinc must be constantly dampened by zinc homeostatic proteins such as ZNT and MT. Notably, ZNT1 and certain MTs are considered to contribute substantially to this homeostatic process because their expression is regulated in a coordinated manner by the extracellular zinc status^31–33^, with the genes encoding these proteins being targets of metal response element-binding transcription factor 1 (MTF1)^17,32,34^. Moreover, we recently reported that the cell-surface expression of ZNT1 is elaborately regulated independently of MTF1^18^. These lines of evidence support the notion that ZNT1 and MT respond to changes in ZIP expression, specifically to both increased and decreased expression of ZIPs. However, the mechanism underlying this response has not been experimentally examined at the molecular level.

In this study, we aimed to clarify how ZNT1 and MT expression responds to alterations in ZIP expression under normal cell-culture conditions in the absence of zinc supplementation. We used the human osteosarcoma cells, U2OS, for the transient transfection of ZIPs (specifically, ZIP4, ZIP5, ZIP7, and ZIP13) and the Madin–Darby canine kidney (MDCK) cells harboring the FLp-In™ T-Rex for the stable transfection of ZIPs (specifically, ZIP4, ZIP5, and ZIP7). The Tet-regulatable promoter in the MDCK cells enables induction of ZIPs through doxycycline (Dox) treatment. The expression of the ZIPs could be controlled through the presence or absence of Dox. Using these systems, we examined whether the expression of both ZNT1 and MT is sophisticatedly regulated by the expression status of ZIPs, in particular, that of the cell-surface-localized ZIPs, such as ZIP4 and ZIP5^6,7,35–37^. We further examined whether an increase in the expression of the apically localized ZIP4 promoted the expression of the basolaterally localized ZNT1 in polarized cells; this mechanism is expected to be operative in enterocytes because ZIP4 and ZNT1 play critical roles in zinc absorption in these cells. These findings provide novel molecular insights into how zinc homeostasis is maintained by the zinc homeostatic proteins when ZIP expression is altered by physiological and pathological stimuli.

## Results

### Cell-surface ZNT1 expression is enhanced by doxycycline (Dox)-induced expression of ZIP4 and ZIP5

**Figure 1:**
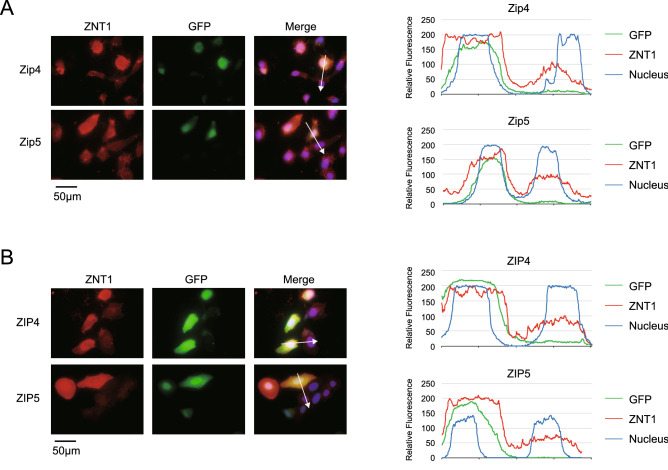
Cell-surface ZNT1 expression is enhanced by Dox-induced expression of mouse Zip4 and Zip5 and human ZIP4 and ZIP5. (**A,B**) Transiently expressed Zip4/Zip5 and ZIP4/ZIP5 enhanced cell-surface ZNT1 expression. U2OS cells were transiently transfected with an IRES-GFP expression plasmid harboring Zip4-HA or Zip5-HA cDNA (**A**) or ZIP4-HA or ZIP5-HA cDNA (**B**), and transfected cells were identified by their GFP fluorescence. DAPI staining is also shown in the merged images, where line-profile analysis was used to semi-quantitatively determine relative fluorescence intensity (white arrows); the graphs on the *right* show the relative fluorescence intensity along each arrow. Each experiment was performed at least thrice, and representative results from independent experiments are shown.

MT expression is reported to be upregulated following the overexpression of ZIPs^22,38^, but whether ZNT1 expression is also affected by ZIP overexpression has remained unexamined. Therefore, we determined how ZNT1 expression responds to the overexpression of ZIPs that localize on the cell surface, specifically ZIP4 and ZIP5. We first measured ZNT1 expression in U2OS cells transiently transfected with expression plasmids carrying ZIP4 and ZIP5 cDNA. We generated mouse Zip4 and Zip5, and their human counterparts, ZIP4 and ZIP5, as fusion proteins tagged with influenza hemagglutinin (HA) at the C-terminus. All cDNAs were subcloned into the internal ribosome entry site (IRES)-GFP plasmid. Immunofluorescence staining revealed that the overexpression of either Zip4 or Zip5, whose cellular expression was confirmed based on GFP fluorescence, induced cell-surface expression of ZNT1 (Fig. 1A). We confirmed the consistency of the responses by overexpressing their human counterparts, because there are several differences between Zip4 and ZIP4 in their zinc sensing mechanism and their expression pattern in AE-causing mutations (*e.g.*, P200L)^3,39–42^; The cell-surface ZNT1 expression showed a similar increase when ZIP4 or ZIP5 was transiently expressed in U2OS cells (Fig. 1B).

### ZNT1 and MT expression is increased in a coordinated manner in response to Dox-induced expression of Zip4/Zip5 or ZIP4/ZIP5

**Figure 2:**
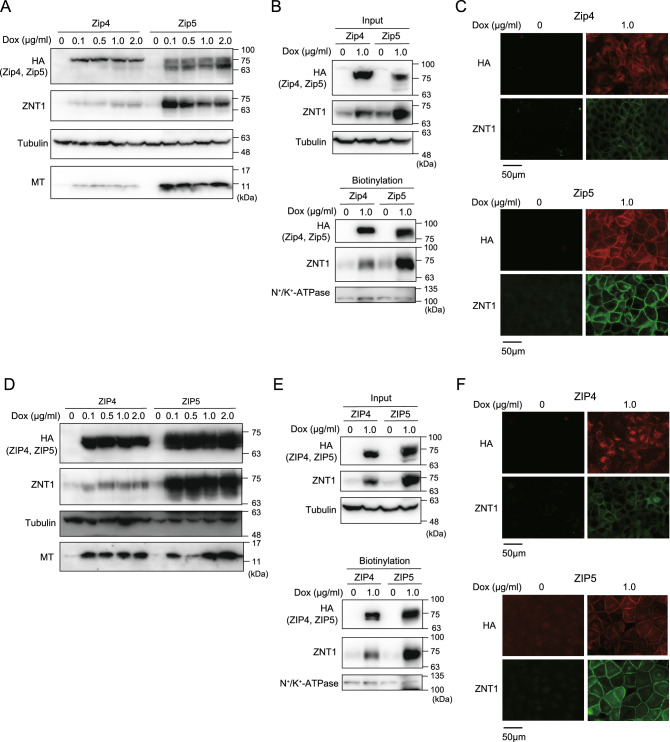
ZNT1 and MT expression increases in a coordinated manner in response to Dox-induced expression of Zip4/Zip5 or ZIP4/ZIP5. (**A**) Upregulated expression of ZNT1 and MT accompanied Dox-induced expression of Zip4/Zip5 in MDCK cells. MDCK cells stably expressing Zip4 or Zip5 were cultured with the indicated concentrations of Dox for 24 h, and then ZNT1 and MT expression was examined. Tubulin was used as a loading control. (**B,C**) ZNT1 accumulated on the cell surface upon Dox-induced Zip4/Zip5 expression. MDCK cells cultured with or without 1.0 μg/mL Dox for 24 h were used in cell-surface biotinylation assays (**B**) or subject to immunofluorescence staining (**C**). In (**B**) tubulin and Na^+^/K^+^-ATPase were used as the loading control for input and biotinylation, respectively. (**D–F**) Same experiments as in (**A–C**) but performed using ZIP4 and ZIP5. Each experiment was performed at least thrice, and representative results from independent experiments are shown.

To closely examine how ZNT1 expression is affected by ZIP4 and ZIP5, we established MDCK cells stably expressing mouse Zip4 and Zip5 and human ZIP4 and ZIP5, with the expression of the exogenous proteins being under the control of Dox treatment. In MDCK cells stably expressing mouse Zip4 or Zip5, Dox treatment (final conc. 0.1–2.0 μg/mL) for 24 h induced the expression of Zip4 or Zip5 (Fig. 2A), and this increased expression was accompanied by the upregulated expression of both ZNT1 and MT (Fig. 2A); the observed effect was specific because no increased expression was detected in parental MDCK cells (Suppl. Fig. 1). Moreover, treatment with Dox (1.0 μg/mL) induced the accumulation of Zip4/Zip5 on the cell surface, which increased the cell-surface expression of ZNT1 (Fig. 2B,C); this is in accord with our transient transfection studies on U2OS cells (Fig. 1). Moreover, ZNT1 and MT expression was similarly upregulated after Dox treatment in MDCK cells stably expressing human ZIP4 or ZIP5 (Fig. 2D), with ZNT1 again accumulating on the cell surface (Fig. 2E,F). Intriguingly, ZNT1 and MT expression was more potently induced in cells expressing Zip5 or ZIP5 than Zip4 or ZIP4, which also agrees with the results of the transient transfection experiments on U2OS cells (Fig. 1). However, the induced ZIP4 expression stimulated MT expression as potently as did ZIP5 expression (Fig. 2D).

### Potent induction of ZNT1 expression mediated by Zip5 is associated with Zip5 cytosolic variable loop

**Figure 3:**
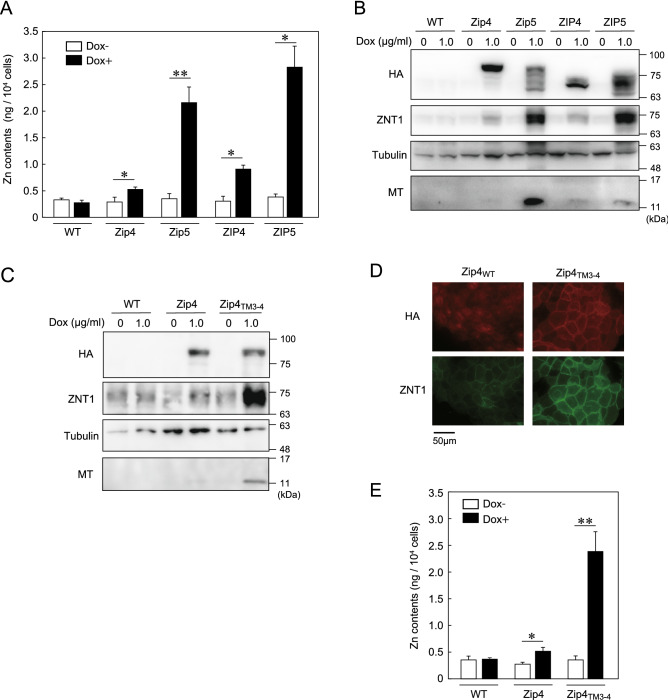
Potent induction of ZNT1 expression mediated by Zip5 is associated with Zip5 cytosolic variable loop. (**A**) Zinc content measured using ICP-MS analysis. Parental MDCK cells and MDCK cells stably expressing Zip4-HA, Zip5-HA, ZIP4-HA, or ZIP5-HA were cultured with 1.0 μg/mL Dox for 24 h and then collected for ICP-MS analyses (n = 3). (**B**) Immunoblotting performed using cell lysates prepared from the indicated cells cultured as in (**A**). (**C**) ZNT1 and MT expression induced by Zip4_TM3–4_-HA mutant was higher than that induced by Zip4. Parental MDCK cells and MDCK cells stably expressing Zip4-HA or Zip4_TM3–4_-HA were cultured with 1.0 μg/mL Dox for 24 h. (**D**) Immunofluorescence labeling for confirming enhanced cell-surface expression of ZNT1 in MDCK cells stably expressing Zip4_TM3–4_-HA; the cells were cultured as in (**C**). (**E**) Zinc content, measured using ICP-MS analysis, in indicated MDCK cells cultured as in (**C**) (n = 3). In (**B,C**), tubulin was used as the loading control. Each experiment was performed at least thrice, and representative results from independent experiments are shown.

We hypothesized that the upregulation of ZNT1 and MT expression was caused by an increase in the cytosolic zinc concentration mediated by ZIP4 and ZIP5 following Dox-induced expression. We examined this possibility and also investigated why ZNT1 and MT expression was induced more strongly by Zip5 and ZIP5 than by Zip4 and ZIP4. We cultured MDCK cells expressing each of the four proteins in the presence or absence of Dox (1.0 μg/mL) and then performed inductively coupled plasma-mass spectrometry (ICP-MS) analysis; the ICP-MS measurements showed that Dox-induced expression of Zip4/ZIP4 and Zip5/ZIP5 increased the cellular zinc content, and that Zip5 and ZIP5 more potently increased the zinc content than did Zip4 and ZIP4 (Fig. 3A). Under this culture condition, in which all the ZIPs were expressed at comparable levels, ZNT1 and MT expression was more strongly induced by Zip5 and ZIP5 than by Zip4 and ZIP4 (Fig. 3B). To comprehensively investigate the differences between ZIP4 and ZIP5, we constructed chimeric mutants of Zip4 and Zip5 by swapping their cytosolic variable loop between transmembrane (TM) helices III and IV; specifically, we constructed Zip4_TM3–4_ and Zip5_TM3–4_ (Fig. 3C, Suppl. Fig. 2). Dox-induced expression of Zip4_TM3–4_, similar to Zip5 expression, more potently increased ZNT1 and MT expression than did Zip4 expression (Fig. 3D), which was reflected by the increased cell-surface expression of ZNT1 (Fig. 3D). However, we were unable to evaluate the effect of Zip5_TM3–4_ because cell-surface expression of the protein was impaired (Suppl. Fig. 2). Moreover, ICP-MS measurements showed that after Dox treatment, the zinc content was higher in cells expressing Zip4_TM3–4_ than in Zip4-expressing cells (Fig. 3E), which can explain the potent induction of ZNT1 and MT expression by Zip5. These results indicate that the cytosolic variable loop of Zip5 is responsible for the higher zinc-transport activity of Zip5 than Zip4, and further suggest that the ZIP-mediated expression of ZNT1 and MT is distinctly and specifically regulated by different ZIPs.

### Expression alteration of intracellularly localized ZIPs does not substantially alter ZNT1 and MT expression

**Figure 4:**
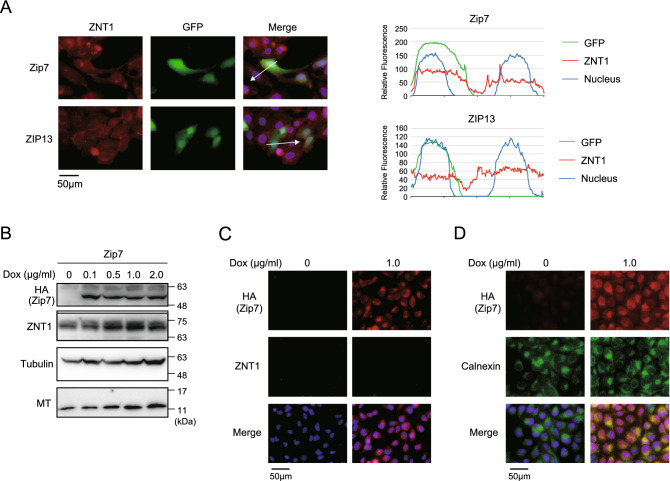
Expression alteration of intracellularly localized ZIPs does not substantially alter ZNT1 and MT expression. (**A**) Transiently expressed mouse Zip7 and human ZIP13 did not enhance cell-surface ZNT1 expression. U2OS cells were transiently transfected with an IRES-GFP expression plasmid harboring Zip7-HA or ZIP13-HA cDNA and then examined as described in Fig. 1. Merged images with DAPI staining are also shown, and the *right* graphs show the relative fluorescence intensity determined semi-quantitatively using line-profile analysis. (**B,C**) Expression of ZNT1 and MT was only slightly altered by Dox-induced expression of Zip7 in MDCK cells. MDCK cells stably expressing Zip7 were cultured with the indicated concentrations of Dox for 24 h in (**B**) or with 1.0 μg/mL Dox in *C*, and ZNT1 and MT expression levels were examined through immunoblotting (**B**) and immunofluorescence staining (**C**). In (**B**), tubulin was used as the loading control. (**D**) Confirmation of ER localization of induced Zip7. MDCK cells were cultured with or without 1.0 μg/mL Dox for 24 h and then immunofluorescence staining was performed; concurrent immunostaining of the ER marker calnexin was used to assess Zip7 subcellular localization. Each experiment was performed at least thrice, and representative results from independent experiments are shown.

Several ZIPs localize to intracellular compartments and release zinc from the lumen of the compartments into the cytosol^10–14^. Thus, we next examined how altering the expression of intracellularly localized ZIPs affects ZNT1 and MT expression. Transient transfection experiments were performed using the IRES-GFP plasmid carrying cDNA encoding mouse Zip7 and human ZIP13, which localize to the ER or Golgi apparatus, respectively, and the results showed that Zip7 or ZIP13 expression only minimally affected ZNT1 expression (Fig. 4A). To further confirm these minor effects on ZNT1 and MT expression, we established MDCK cells stably expressing Zip7 (as in the aforementioned experiments) and examined the effect of Dox-induced Zip7 expression on ZNT1 and MT expression. In contrast to what was observed in cells expressing Zip4/ZIP4 and Zip5/ZIP5, Dox-induced expression of Zip7 in the ER again only slightly increased the expression of ZNT1 and MT (Fig. 4B–D). These results showed that changes in the expression of intracellularly localized ZIPs produce only minor effects on ZNT1 and MT expression as compared with alterations in the expression of cell-surface ZIPs.

### Elimination of the induced expression of ZIPs results in the decreased expression of ZNT1 but not of MT

**Figure 5:**
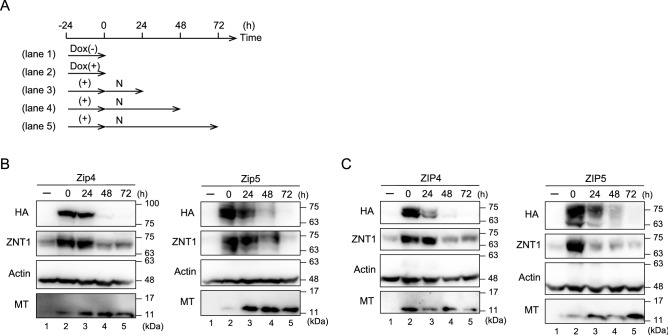
Elimination of the induced expression of ZIPs results in a reduced expression of cell-surface ZNT1. (**A**) Schematic depicting the assay time course. MDCK cells stably expressing Zip4, Zip5, ZIP4, or ZIP5 were cultured with or without 0.1 μg/mL Dox for 24 h, and then the cells were cultured in normal medium (N) for an additional 24–72 h after washing thrice with PBS. (**B,C**) ZNT1 expression upregulated by Dox-induced expression of Zip4 or Zip5 (**B**) or ZIP4 or ZIP5 (**C**) decreased after Dox removal. However, a marked reduction of MT expression did not accompany the reduction of ZNT1 expression. Actin was used as the loading control. Each experiment was performed at least thrice, and representative results from independent experiments are shown.

If ZNT1 and MT play pivotal roles as zinc homeostatic proteins, a decrease in the expression of both proteins would be expected to accompany a reduction in ZIP expression. To test this possibility, we cultured the ZIP-expressing MDCK cells according to the time course shown in Fig. 5A, and we treated these cells with a 1/10 Dox concentration (0.1 μg/mL Dox) as compared with that in previous experiments to ensure rapid elimination of Dox after inducing expression of Zip4/Zip5 (Fig. 5B) or ZIP4/ZIP5 (Fig. 5C). Elimination of Dox after treatment for 24 h (the time point at which Zip4/Zip5 expression was highly induced) gradually decreased the expression of Zip4/ZIP4 and Zip5/ZIP5 every 24 h up to 72 h (Fig. 5B,C). Notably, the decrease in the expression of Zip4/ZIP4 and Zip5/ZIP5 was accompanied by diminished expression of ZNT1 (Fig. 5B,C), which clearly indicates that ZNT1 expression responds in a sophisticated manner to a reduction in the expression of Zip4/ZIP4 and Zip5/ZIP5. However, during the time course of this assay, MT expression did not change substantially (Fig. 5B,C). These results indicate that cellular zinc homeostatic maintenance is accomplished by zinc homeostatic proteins such as ZNT1 and MT when ZIP expression is altered, although the expression of ZNT1 and MT is not invariably controlled in the same manner in these cellular responses.

### Increased expression of apically localized ZIP4 promotes the expression of basolaterally localized ZNT1

**Figure 6:**
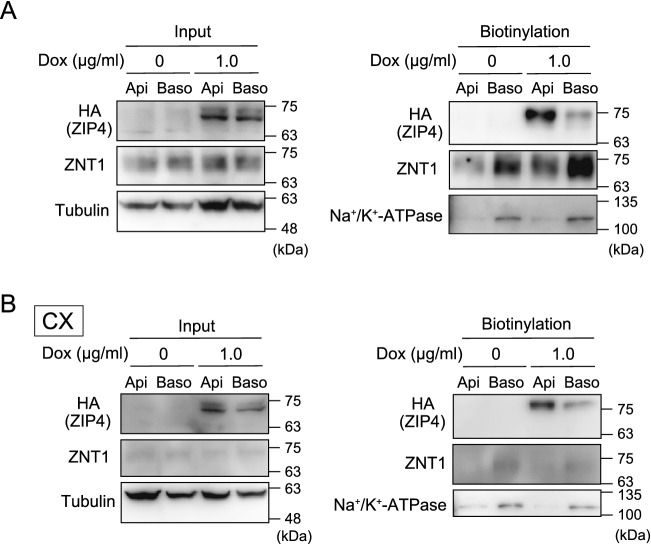
Increased expression of apically localized ZIP4 induces the expression of basolaterally localized ZNT1. (**A**) MDCK cells stably expressing ZIP4-HA and grown in transwell plates were treated with Dox for 24 h. Next, the biotinylation reagent sulfo-NHS-SS-biotin was added to either the apical (Api) or basolateral (Baso) compartment of the transwell plate. Lastly, solubilized proteins captured using streptavidin beads were analyzed by immunoblotting with specific antibodies. (**B**) Same experiments as in (**A**), performed similarly but employing a zinc-deficient medium generated using Chelex-100 resin-treated FCS (CX). In (**A,B**), input refers to aliquots of the biotinylated proteins before avidin capture, and biotinylation refers to avidin-captured proteins. Tubulin and Na^+^/K^+^-ATPase were used as the loading control for the input and for monitoring the efficiency of basolateral membrane biotinylation, respectively.

Lastly, we investigated the physiological importance of the coordinated expression of ZIPs and ZNT1. Polarized MDCK cells are widely used for studying vectorial transcellular transport from the apical side to the basolateral side, we thus used these cells and focused on the coordinated expression of ZIP4 and ZNT1, which is considered to be essential in the zinc-absorption process in enterocytes^43–46^; in these cells, ZIP5 is basolaterally localized^7,35,37^. No study to date has presented direct experimental evidence of such a coordinated expression of the proteins. Specifically, we examined how induction of ZIP4 expression on the apical surface affects ZNT1 expression on the basolateral surface by using polarized MDCK cells expressing ZIP4; we used these cells as extracellular zinc was taken up more efficiently by ZIP4 than Zip4 (Figs. 2, 3). The polarized MDCK cells were grown on transwell plates and treated with Dox (1.0 μg/mL) from both the apical side and the basolateral side for 24 h. Subsequently, a biotinylation reagent was added to either the apical or basolateral compartment of the transwell plates after washing, and the biotinylated proteins were then extracted in lysis buffer and captured using streptavidin beads. Whereas ZIP4 was detected as a biotinylated apical protein, ZNT1 was detected as a biotinylated basolateral protein, with the protein expression being increased upon Dox treatment (Fig. 6A). However, no increase in ZNT1 expression was detected following Dox treatment when the polarized MDCK cells were cultured in a zinc-deficient culture medium containing Chelex-treated fetal calf serum (FCS; CX in Fig. 6B), which indicates that the zinc uptake mediated by ZIP4 is responsible for the upregulated expression of ZNT1. These results indicate that apically induced ZIP4 expression caused basolateral ZNT1 expression, and thus suggest that ZIP4-driven ZNT1 expression is operative in polarized cells, which is essential for vectorial transcellular transport in zinc absorption.

## Discussion

**Figure 7:**
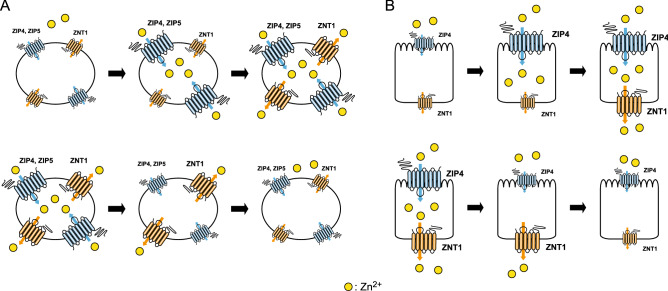
Model depicting ZIP-driven ZNT1 expression for homeostatic control. (**A**) Model showing ZIP-driven ZNT1 expression in generic cells. Increased expression of cell-surface ZIPs results in increased zinc content in the cytosol, which leads to the enhanced expression of ZNT1 and the subsequent dampening of increases in cytosolic zinc concentration (*upper*). Conversely, decreased expression of cell-surface ZIPs causes a decrease in the zinc content in the cytosol, which results in the decreased expression of ZNT1 and thus the maintenance around a homeostatic setpoint at a specific concentration in each cell type (*lower*). (**B**) Prediction model showing ZIP4-driven ZNT1 expression in the zinc-absorption process in enterocytes. Increased ZIP4 expression on the apical surface results in enhanced zinc content in the cytosol, which, in turn, leads to the upregulation of ZNT1 expression and thereby enables efficient export of zinc on the basolateral side into the blood stream (*upper*). Conversely, decreased ZIP4 expression on the apical surface causes the opposite responses through a decrease in the zinc content in the cytosol and decreased ZNT1 expression, and thus leads to reduced zinc export (*lower*).

Homeostatic responses of ZNT1 and MT to high zinc concentrations have been proposed as zinc “buffering and muffling” mechanisms that can adjust and maintain the cytosolic zinc concentration around a homeostatic setpoint^4,20,21,47^. These mechanisms are expected to function when ZIP expression is induced by various stimuli. Conversely, ZNT1 and MT expression must be repressed to prevent the loss of cellular zinc when ZIP expression is decreased, to maintain the zinc concentration around a homeostatic setpoint (Fig. 7A). Our results here indicate that this mechanism operates in cells under physiological conditions by adjusting ZNT1 and MT expression in response to changes in ZIP expression. Notably, in our experiments, Dox-induced ZIP4/ZIP5 expression or elimination of their induced expression was performed in the absence of zinc supplementation. To the best of our knowledge, this is the first experimental report describing coordinated variations in ZNT1 and MT expression in response to the alteration of ZIP expression under physiological zinc concentrations. ZNT1 and MTs are ubiquitously expressed; therefore, their expression responses could be observed in various types of cells in vitro and in vivo.

*ZNT1* and *MT* expression has been shown to be regulated in a coordinated manner in several previous studies^18,32,48^. Moreover, *apo*-MT is reported to be more rapidly proteolyzed than *holo*-MT^49–51^, and partially metalated MT is reported to be more stable than *ap*o-MT^52^. In this study, we observed differential regulation of ZNT1 and MT expression, with ZNT1 but not MT being degraded when the induced ZIP expression was eliminated (Figs. 2, 5); this could be explained by the differences in the MT metalation status. The conformation of MT becomes progressively more ordered during metalation as the metal numbers are increased (up to 7)^53^. Thus, if metalated MT loses the metalated zinc singly in response to the loss of ZIP expression, MT would become resistant to degradation until *apo*-MT is formed in cells. However, further investigation is required to determine whether partially metalated forms of MT show distinct degradation rates^5^. By contrast, ZNT1 expression might be regulated by changes in zinc concentrations because ZNT1 was degraded under the same conditions. This possibility is potentially supported by the results showing that both ZNT1 and MT were degraded when MDCK cells were treated for 6 h with a zinc chelator, N,N,N',N'-tetrakis(2-pyridylmethyl)ethylenediamine (TPEN), to rapidly cause zinc deficiency while maintaining Zip4 and Zip5 expression (Suppl. Fig. 3). These results suggest that the balance between the activities of ZIPs and ZNT1 could affect MT turnover rates in cells. Clarifying the molecular mechanism underlying this phenomenon would enhance our understanding of the control of cellular zinc homeostasis, and the mechanism thus warrants elucidation in future studies.

In our MDCK cell system, Zip5 and ZIP5 increased the cellular zinc content and thereby induced ZNT1 and MT expression more potently than did Zip4 and ZIP4, and this difference can be attributed to the cytosolic variable loop of Zip5. We have not yet uncovered the mechanistic basis for this unique property of the loop in ZIP5, but one possibility is that the variable loop in ZIP5 might associate with unknown specific proteins, which might, in turn, facilitate the zinc-transport ability of ZIP5. The first half of the variable loop in ZIP4 is rich in His residues, whereas that in ZIP5 is rich in Arg residues^35^, which might account for the difference. As compared with the molecular features of ZIP4, which have been clarified to a considerable extent^38–40,42,54–57^, those of Zip5 remain poorly described^8^, in contrast to the elucidation of the physiological relevance of the protein^9,58^. Thus, clarifying this matter will provide crucial information regarding ZIP activity and function.

ZNT1 and MT expression was only slightly upregulated upon induction of the expression of two intracellularly localized ZIPs, Zip7 and ZIP13. By contrast, induction of the expression of cell-surface-localized ZIPs, such as ZIP4 and ZIP5, increased the expression of cell-surface ZNT1 and cytosolic MT. These differences are intriguing because both types of ZIPs have been shown to play crucial roles in zinc signaling^1,59^, probably by mediating zinc fluxes in the cytosol. Thus, this finding could provide valuable insights regarding how zinc signaling is triggered. For example, zinc signaling stimulated by intracellularly localized ZIPs might require an as yet unknown specific mechanism (perhaps involving unidentified chaperone proteins) to trigger zinc signaling. Alternatively, the target protein for zinc signaling might exist close to the intracellularly localized ZIPs to ensure efficient transmission of the signal. We have not yet obtained data indicating this possibility, and further investigation is required to clarify this point.

Another interesting finding reported here is that enhanced expression of apically localized ZIP4 resulted in induced expression of basolaterally localized ZNT1 in polarized cells; the mechanism underlying this regulated expression is expected to be operative in zinc absorption in enterocytes (Fig. 7B). However, we used non-intestinal MDCK cells to perform our experiments. Considering that ZIP4 expression is sophisticatedly regulated by the zinc status in cells^38–40,54,55^, and further that ZNT1 expression is driven by the zinc uptake mediated by ZIP4, ZIP4-driven ZNT1 expression could serve as a critical mechanism to tightly control the zinc-absorption rate of cells. The regulation of cell-surface ZNT1 expression at the posttranscriptional level (independent of MTF1)^18^ would enable the prompt response required to maintain cellular zinc homeostasis after rapid alteration of ZIP4 expression, which, in turn, would help achieve systemic zinc homeostatic maintenance.

In conclusion, the expression of ZNT1 and MT responds in a sophisticated manner to alterations in the expression of cell-surface ZIPs. This coordinated expression response is likely to be critical for maintaining cellular zinc homeostasis under physiological and pathological conditions. Moreover, the ZIP4-driven ZNT1 expression mechanism reported here to function in polarized cells is expected to be operative in zinc absorption in enterocytes, with ZIP4 and ZNT1 being involved in the process. Our findings provide crucial insights into how systemic and cellular zinc homeostasis is maintained around a homeostatic setpoint.

## Materials and methods

### Cell culture and stable or transient transfection

MDCK cells harboring the FLp-In™ T-Rex system (MDCK cells in this study)^60^ and U2OS cells harboring a Tet-on system (U2OS cells in this study) (Takara Bio Inc., Otsu, Japan) were maintained at 37 °C in a humidified 5% CO_2_ incubator in Dulbecco’s modified Eagle’s medium (DMEM) (FUJIFILM Wako Pure Chemical Corporation, Osaka, Japan) containing 10% (v/v) heat-inactivated FCS (Biosera, Kansas City, MO, USA) and 100 U/mL penicillin and 100 μL/mL streptomycin (Nacalai Tesque, Kyoto, Japan), as described previously^61^. To establish MDCK cells stably expressing Zip4-HA, Zip5-HA, Zip4_TM3–4_-HA, Zip5_TM3–4_-HA, ZIP4-HA, ZIP5-HA, or Zip7-HA, an expression plasmid (pcDNA5/FRT/TO) containing the specific cDNA was co-transfected with pOG44 plasmid at a 9:1 ratio by using Lipofectamine 2000 reagent (Thermo Fisher Scientific, Waltham, MA, USA). Stable clones were selected in medium containing 250–500 μg/mL hygromycin B (Nacalai Tesque) for 3 weeks. U2OS cells were transiently transfected with 0.5 μg of empty IRES-GFP plasmid or IRES-GFP plasmid harboring each cDNA by using Lipofectamine 2000 reagent; complete culture medium was then added at 8 h post-transfection, and the cells were cultured for an additional 24 h before use in experiments. For studies on polarized MDCK cells, MDCK cells were cultured on 24 mm polyester-membrane transwell plates (pore size: 0.4 μm; Greiner Bio-One, Frickenhausen, Germany) to allow the formation of tight junctions. To generate a zinc-deficient culture medium, FCS was treated with Chelex-100 resin (CX; Bio-Rad Laboratories, Hercules, CA, USA), as described previously^62^. The zinc chelator TPEN was also used for preparing zinc-deficient cultures, as described previously^18^.

### Plasmid construction

Each ZIP and Zip cDNA was fused with an HA-tag at the C-terminus by using PCR and inserted into the plasmid pcDNA5/FRT/TO (Thermo Fisher Scientific) or IRES-GFP (provided by Dr. Hirohide Saito, Kyoto University). Chimeric mutants of Zip4 and Zip5, in which the cytosolic variable loop between TM helices III and IV was swapped, were constructed using a two-step PCR method as described^63^.

### Immunoblotting analysis

Immunoblotting was performed as described^18^. The cells were cultured in 6-well cell culture plates (Thermo Fisher Scientific). They were washed twice with ice cold PBS and collected with a cell-scraper, centrifuged at 2300×*g* for 5 min at 4 °C. The cells were lysed in NP-40 buffer [50 mM HEPES-HCl (pH 7.4), 100 mM NaCl, 1.5 mM MgCl_2_, 1% Nonidet P-40, and 0.5% deoxycholate] or ALP buffer [10 mM Tris–HCl (pH7.5), 0.5 mM MgCl_2_, 0.1% Triton-X]. The total protein was extracted; 20 μg of the total protein was denatured in 6× SDS sample buffer at room temperature for 30 min, separated using electrophoresis, and transferred to PVDF membranes (Immobilon-P; Millipore Corp., Bedford, MA, USA). Blotted PVDF membranes were blocked with 5% skim milk/0.1% Tween-20 in phosphate-buffered saline (PBS) and then incubated with primary antibodies (diluted in blocking buffer): anti-HA [561] (1:3000; MBL, Nagoya, Japan), anti-MT [1A12] (1:3000; TransGenic Inc., Kobe, Japan), anti-ZNT1 (1:3000)^18^, anti-Na^+^/K^+^-ATPase [sc-28800] (1:3000; Santa Cruz Biochemistry, Santa Cruz, CA, USA), anti-α-tubulin [12G10] (1:3000; deposited to Developmental Studies Hybridoma Bank (DSHB) by J. Frankel and E. M. Nelsen), or anti-actin [JLA20] (1:3000; deposited to DSHB by J.J.-C. Lin). Immunoreactive bands were detected using 1:3000 horseradish peroxidase (HRP)-conjugated anti-mouse or anti-rabbit secondary antibodies (NA931 or NA934, GE Healthcare, Milwaukee, WI, USA) and Immobilon Western Chemiluminescent HRP Substrates (Millipore). Chemiluminescence images were obtained using an ImageQuant LAS 500 system (Cytiva, Marlborough, MA, USA).

### Immunofluorescence staining

MDCK cells cultured on coverslips were fixed with 100% methanol (Nacalai Tesque), stained with anti-ZNT1 (1:3000), anti-HA [HA-11] (1:3000; BioLegend, San Diego, CA, USA), anti-HA [3F10] (1:3000; Roche Molecular Biochemicals, Mannheim, Germany), anti-HA [561] (1:3000; MBL), or anti-calnexin [ADI-SPA-860] (1:3000; Enzo Life Sciences, Plymouth Meeting, PA, USA) primary antibodies, and then stained with appropriate second or third antibodies without permeabilization. U2OS cells cultured on coverslips were fixed with 10% formaldehyde neutral buffer solution (Nacalai Tesque), stained with anti-ZNT1 (1:3000) and anti-GFP [G10362] (1:3000; Thermo Fisher Scientific) antibodies, and then stained with a second antibody after permeabilization with 0.1% Triton X-100. The fluorophore-labeled second and third antibodies used were Alexa 488-conjugated goat anti-mouse IgG, goat anti-rabbit IgG, and rabbit anti-goat IgG, and Alexa 594-conjugated donkey anti-rabbit IgG, donkey anti-mouse IgG, goat anti-mouse IgG, and goat anti-rat IgG (all from Thermo Fisher Scientific). The antibodies were applied at room temperature for 1 h or at 4 °C overnight, and 4,6-diamino-2-phenylindole (DAPI; 1:1000; Abcam) was added during the second and third antibody staining to label nuclei. The stained cells were examined using a fluorescence microscope (FSX100; Olympus, Tokyo, Japan), and images were analyzed using Adobe Photoshop CS. Identical exposure settings and times were used for the corresponding images in each figure. Relative fluorescence intensity was determined semi-quantitatively through line-profile analysis by using cellSens software (Olympus).

### Cell-surface biotinylation assay

Cell-surface biotinylation assays were performed as described previously^38^. After washing cells twice with ice-cold PBS, lysine residues exposed on the extracellular surface were biotinylated by adding EZ-Link, a sulfo-NHS-SS-biotin reagent (Pierce Protein Biology, Thermo Fisher Scientific, Rockford, IL, USA). Subsequently, biotinylated proteins were recovered from streptavidin-coupled beads in 6× sodium dodecyl sulfate (SDS) sample buffer and immunoblotted; an aliquot of the biotinylated proteins before avidin capture (the total cell lysate) was used as the input in immunoblotting. In the case of polarized cells, the cells were washed twice with ice-cold PBS supplemented with 0.1 mM CaCl_2_ and 1.0 mM MgCl_2_, and then the sulfo-NHS-SS-biotin reagent was applied to the apical or basolateral compartment of the transwell plate according to the manufacturer’s instructions (Pierce). After washing twice with PBS, the polyester membrane was excised from the transwell plate by using a scalpel, and the membrane was placed in a 1.5 mL microcentrifuge tube containing 100 μL of NP-40 buffer and sonicated for 20 s. The membrane was removed, and biotinylated proteins were recovered from streptavidin-coupled beads, as described above.

### ICP-MS analysis

Zinc content was determined as described previously^64^. Briefly, cells were washed thrice with PBS containing 1 mM EDTA and collected using sterilized water, and after heating the samples to 180 °C, 60% nitric acid (HNO_3_), 60% perchloric acid, and 30% hydrogen peroxide were added. This procedure was repeated until the removal of all organic materials. After cooling the samples to room temperature, the residues were resuspended in 5 mL of 5% HNO_3_ and the solutions were used for quantifying zinc concentrations by performing ICP-MS (Agilent 7700X/Mass Hunter; Agilent Technologies, Inc., Santa Clara, CA, USA). All tall beakers and sample cups used in this experiment were pretreated with 1% (v/v) HNO_3_ to avoid metal contamination. Standard curves were plotted by preparing 1000 μg/mL (ppm) standard solutions of zinc (FUJIFILM Wako Pure Chemical Corporation) and diluting them in 5% (v/v) HNO_3_ to final metal concentrations of 0, 1, 2, 5, 10, 20, and 50 ng/mL (ppb). For quality control, 1 ng/mL (ppb) of a reference internal standard (indium; In) was measured in parallel with the samples.

### Statistical analysis

All data are expressed as means ± S.D. of triplicate experiments. Statistical significance was determined using Student’s *t* test (comparison of two groups); differences were considered significant at p < 0.05 (*) and p < 0.01 (**).

## Supplementary Information

Supplementary Figures.

## Data Availability

All data generated or analyzed during this study are included in this published article (and its Supporting Information file) or are available from the corresponding author (Taiho Kambe, Kyoto University; E-mail: kambe.taiho.7z@kyoto-u.ac.jp) upon reasonable request.

## Abbreviations

ZIP: Zrt-/Irt-like protein
ZNT: Zn transporter
MT: Metallothionein
MDCK: Madin–Darby canine kidney
Dox: Doxycycline
HA: Hemagglutinin
ICP-MS: Inductively coupled plasma-mass spectrometry
FCS: Fetal calf serum
TPEN: N,N,N′,N′-tetrakis(2-pyridylmethyl)ethylenediamine
