# Supplementary material for: Sophisticated expression responses of ZNT1 and MT in response to changes in the expression of ZIPs
Source: Sci Rep. 2022 May 5;12:7334. doi: 10.1038/s41598-022-10925-2 (PMC9072671; doi:10.1038/s41598-022-10925-2)
Supplement: Supplementary file 1 — Supplementary Figures. [file 41598_2022_10925_MOESM1_ESM.docx]

**Supplementary Information**

**Sophisticated expression responses of ZNT1 and MT in response to changes in the expression of ZIPs**

Shino Nagamatsu^1^, Yukina Nishito^1,2^, Hana Yuasa^1^, Nao Yamamoto^1^, Taiki Komori^1^, Takuya Suzuki^3^, Hiroyuki Yasui^2^, Taiho Kambe^1*^

^1^Division of Integrated Life Science, Graduate School of Biostudies, Kyoto University, Kyoto 606-8502, Japan;

^2^Department of Analytical and Bioinorganic Chemistry, Division of Analytical and Physical Sciences, Kyoto Pharmaceutical University, Kyoto 607-8414, Japan;

^3^Graduate school of integrated sciences for life, Hiroshima University, Higashi-Hiroshima 739-8528, Japan.

**TABLE OF CONTENTS**

**Supplementary Figure 1. ZNT1 and MT expression is not induced by Dox in WT MDCK cells.**

**Supplementary Figure 2. Zip5_TM3-4_ is not trafficked to the cell surface.**

**Supplementary Figure 3. Rapid zinc elimination causes ZNT1 and MT degradation even in the presence of ZIPs.**

**Supplementary Figure 4. Full-length immunoblot images used in Figure 2.**

**Supplementary Figure 5. Full-length immunoblot images used in Figure 3.**

**Supplementary Figure 6. Full-length immunoblot images used in Figure 4.**

**Supplementary Figure 7. Full-length immunoblot images used in Figure 5.**

**Supplementary Figure 8. Full-length immunoblot images used in Figure 6.**

**Supplementary Figure 9. Full-length immunoblot images used in Supplementary Figure 1.**

**Supplementary Figure 10. Full-length immunoblot images used in Supplementary Figure 2.**

**Supplementary Figure 11. Full-length immunoblot images used in Supplementary Figure 3.**

**Supplementary Figure 1. ZNT1 and MT expression is not induced by Dox in WT MDCK cells.** A. Confirmation that ZNT1 and MT expression was not upregulated by Dox treatment in WT MDCK cells. MDCK cells were cultured with the indicated concentrations of Dox for 24 h, and ZNT1 and MT expression levels were examined. Tubulin was used as the loading control.

**Supplementary Figure 2. Zip5_TM3-4_ is not trafficked to the cell surface.** A. Immunoblotting of Zip5 and Zip5_TM3-4_ mutant in MDCK cells. The cells were cultured with or without 1.0 μg/mL Dox for 24 h and then immunoblotting was performed as in Fig. 2. Tubulin was used as the loading control. B. Immunofluorescence staining of Zip5 and Zip5_TM3-4_ mutant. Zip5_TM3-4_ mutant was detected intracellularly and not on the cell surface, as was ZNT1 in these cells.

**Supplementary Figure 3. Rapid zinc elimination causes ZNT1 and MT degradation even in the presence of ZIPs.** MDCK cells stably expressing Zip4 or Zip5 were cultured with or without 0.1 μg/mL Dox for 24 h, and the cells were then cultured with the zinc chelator N,N,N',N'-tetrakis(2-pyridylmethyl)ethylenediamine (TPEN, 10 μM) for an additional 6 h after washing thrice with PBS.


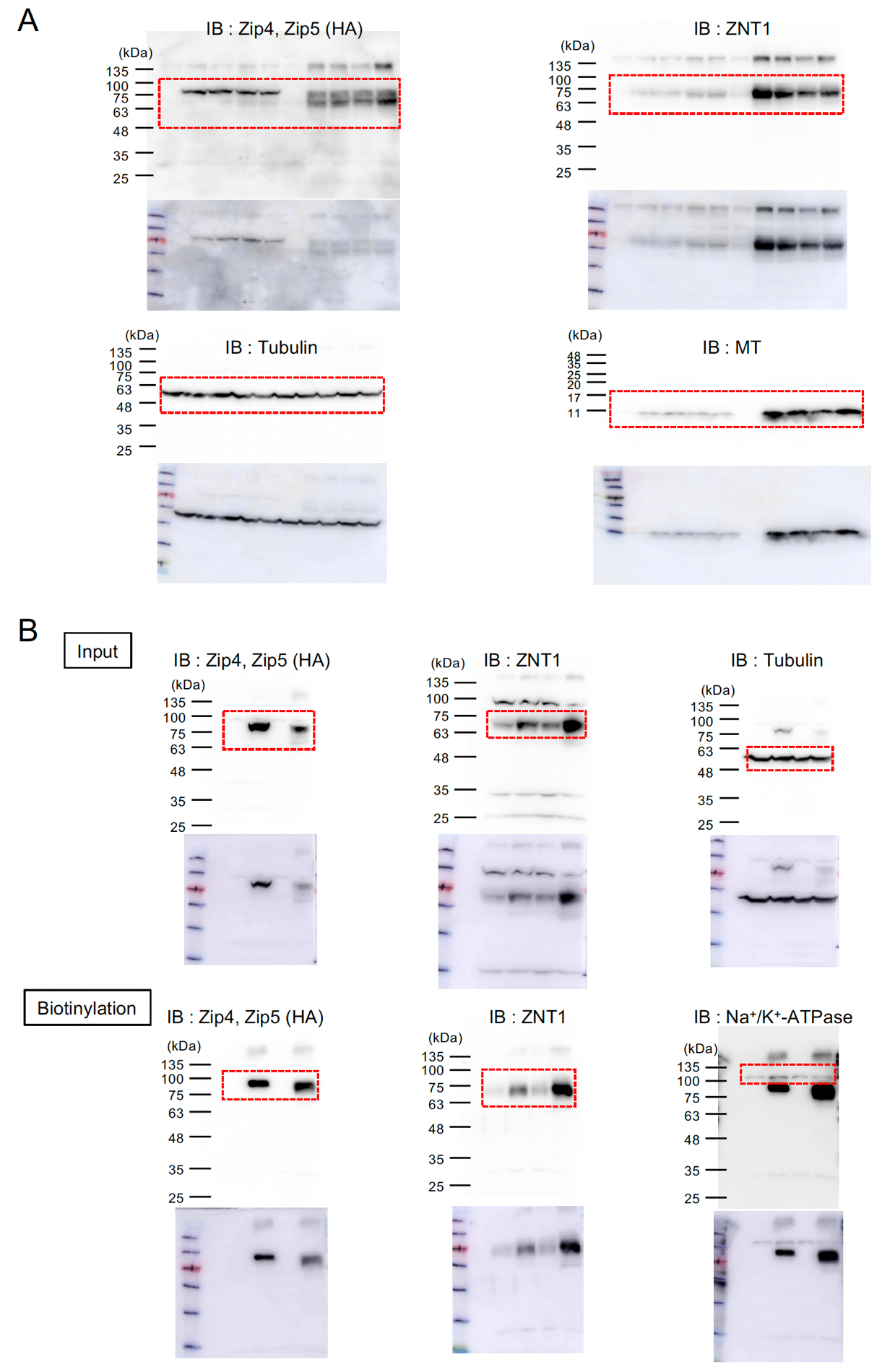


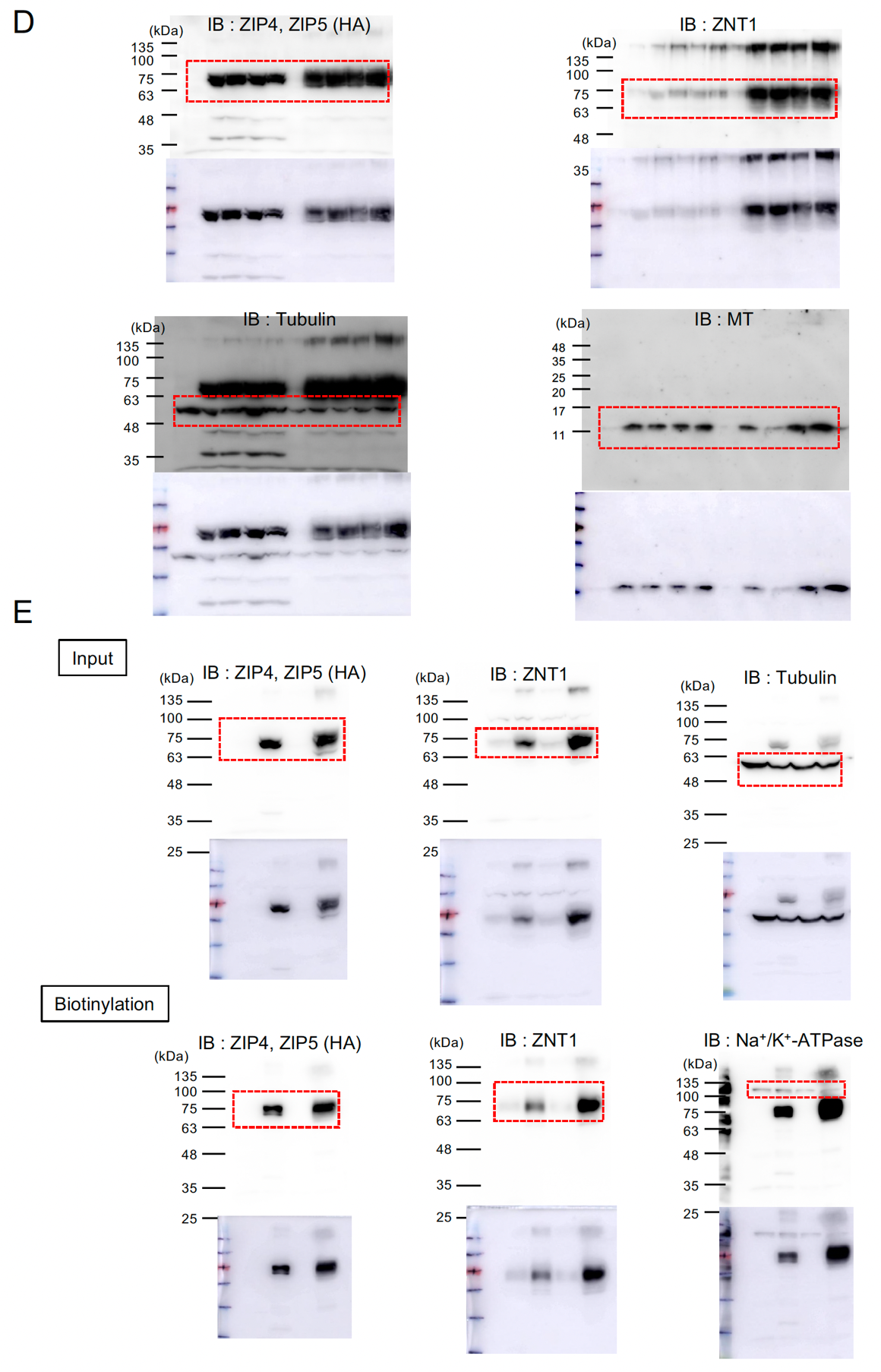


**Supplementary Figure 4. Full-length immunoblot images used in Figure 2.**

The panels used are boxed. The molecular weights of the marker proteins are indicated on the left of the immunoblot images. The images with varying exposures are also shown.


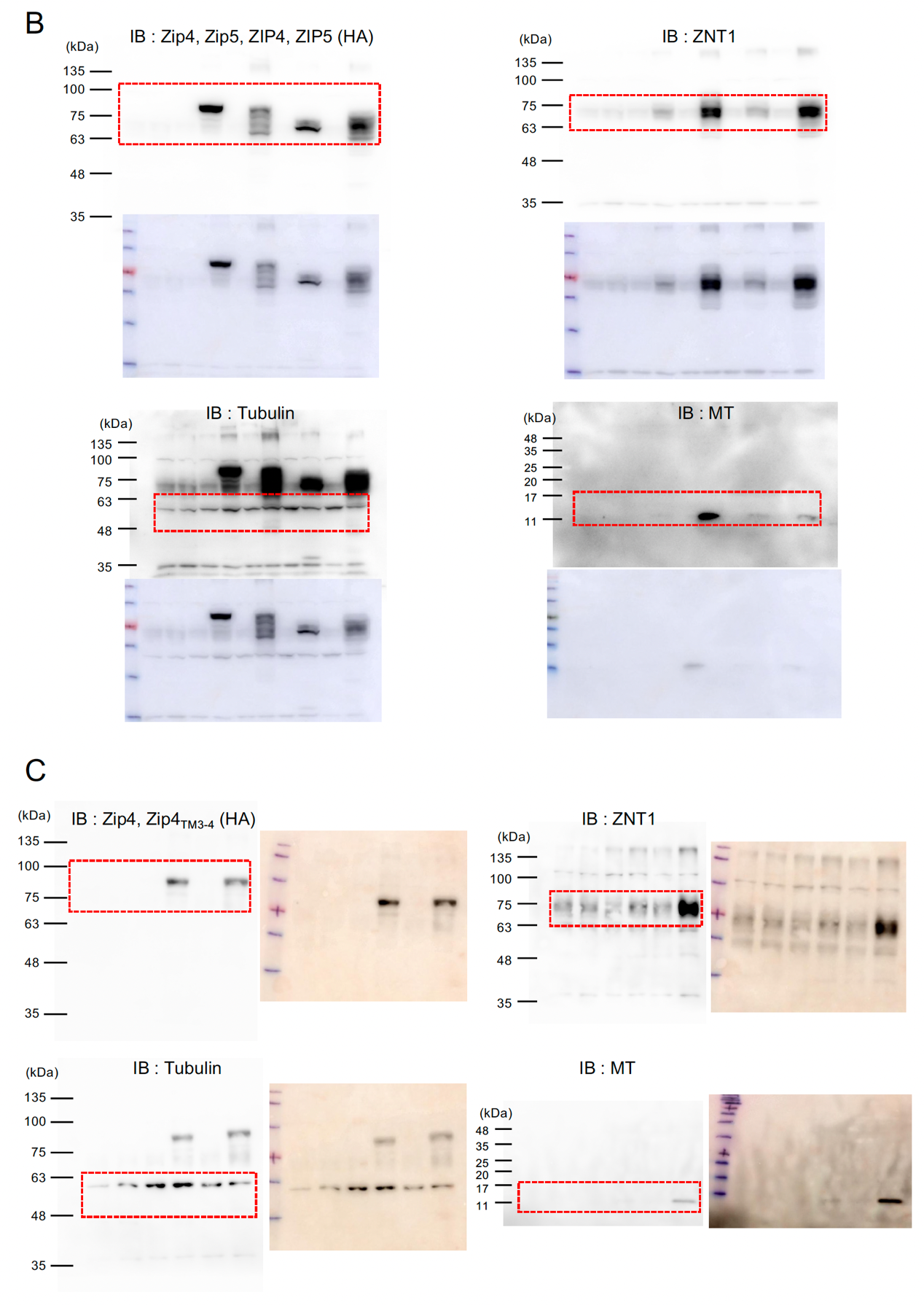


**Supplementary Figure 5. Full-length immunoblot images used in Figure 3.**

The panels used are boxed. The molecular weights of the marker proteins are indicated on the left of the immunoblot images. The images with varying exposures are also shown.


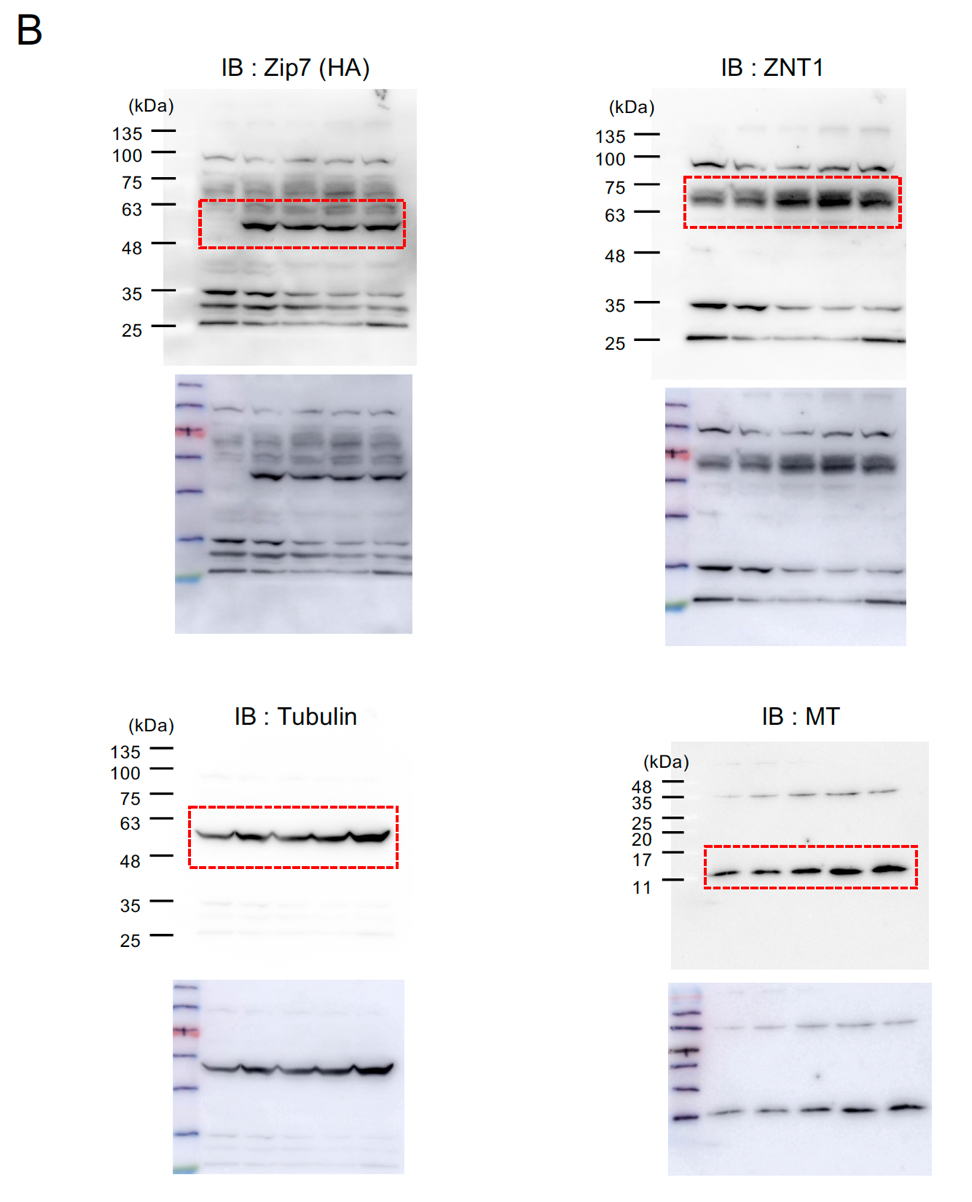


**Supplementary Figure 6. Full-length immunoblot images used in Figure 4.**

The panels used are boxed. The molecular weights of the marker proteins are indicated on the left of the immunoblot images. The images with varying exposures are also shown.

**
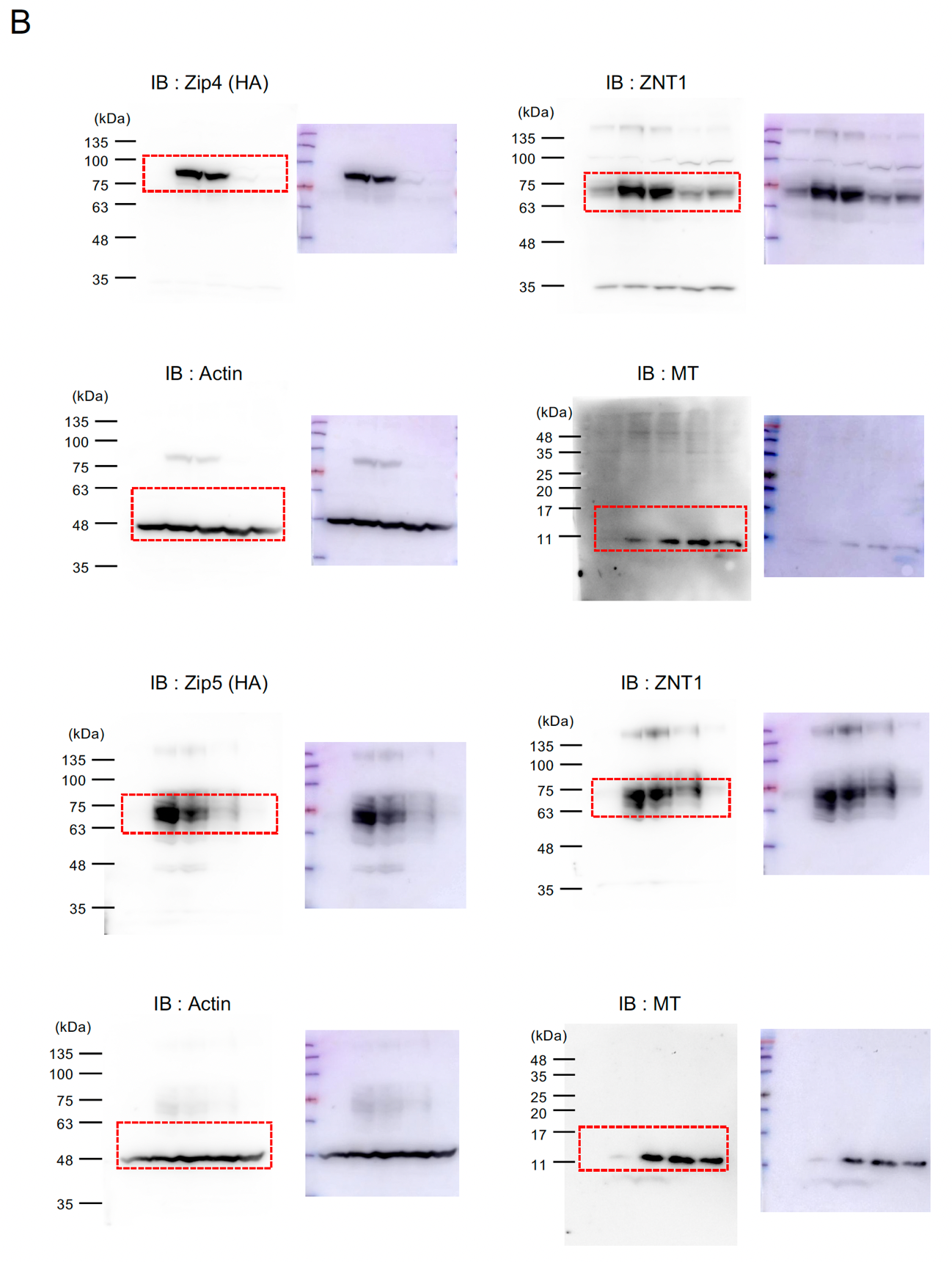
**

**
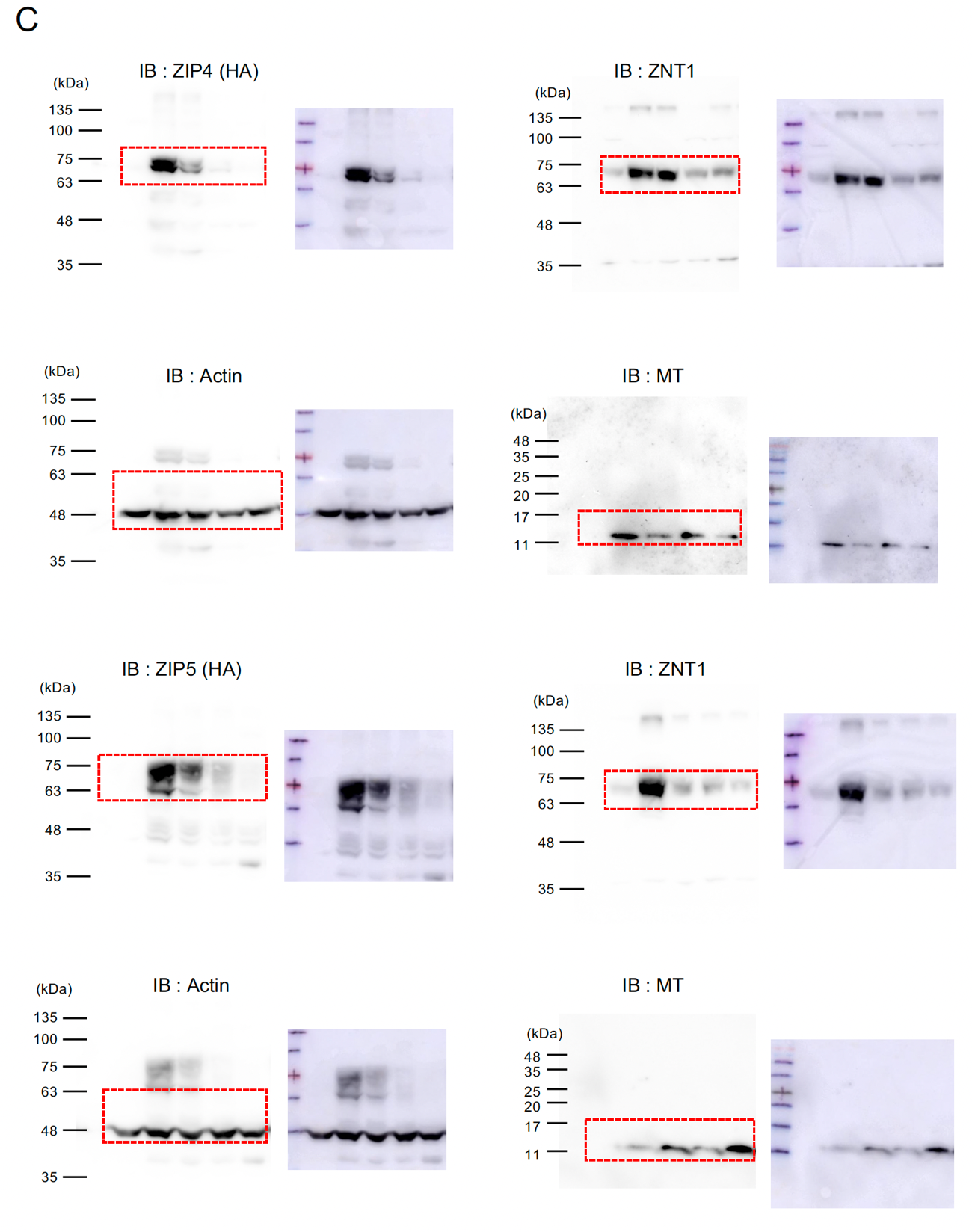
**

**Supplementary Figure 7. Full-length immunoblot images used in Figure 5.**

The panels used are boxed. The molecular weights of the marker proteins are indicated on the left of the immunoblot images. The images with varying exposures are also shown.

**
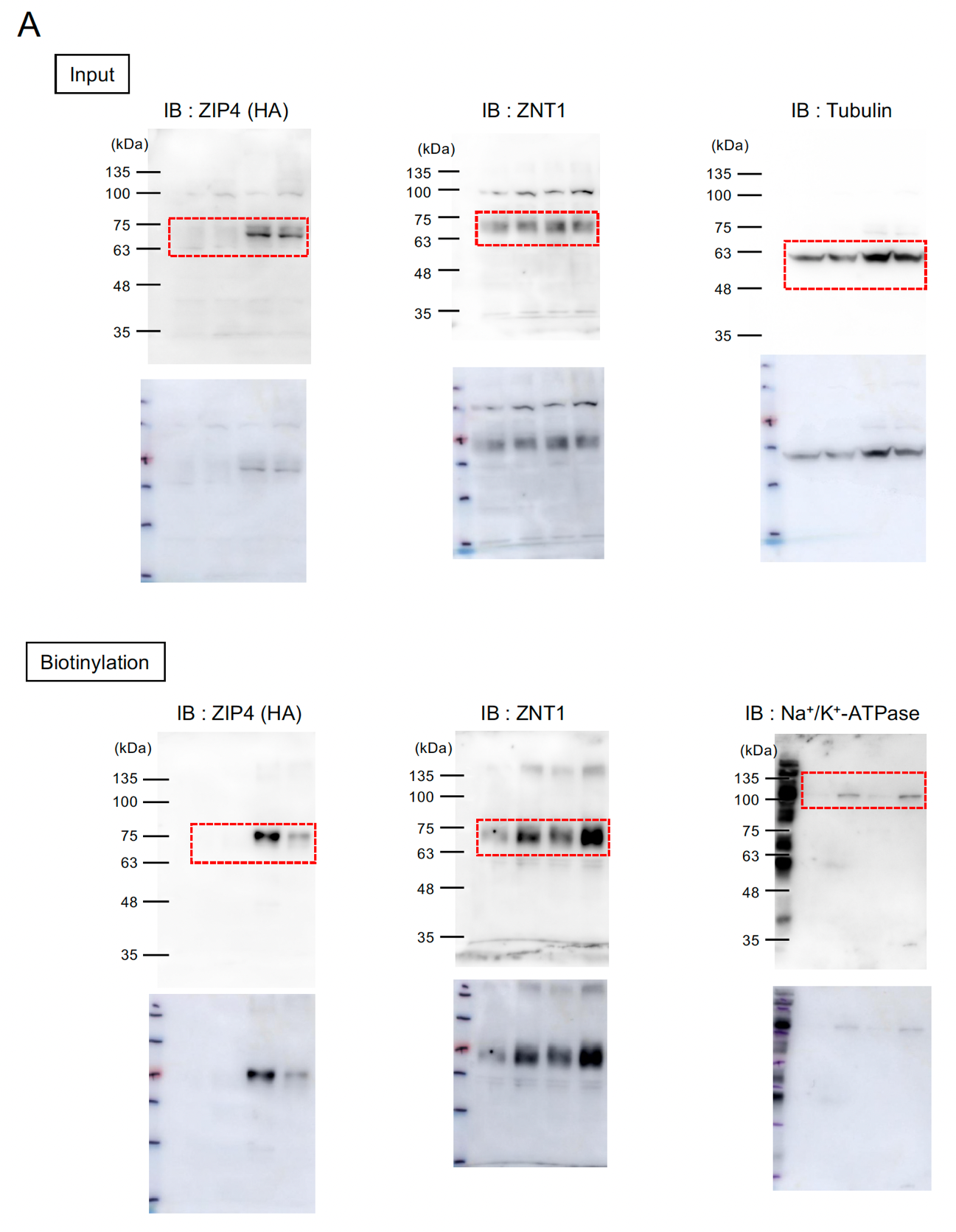
**

**
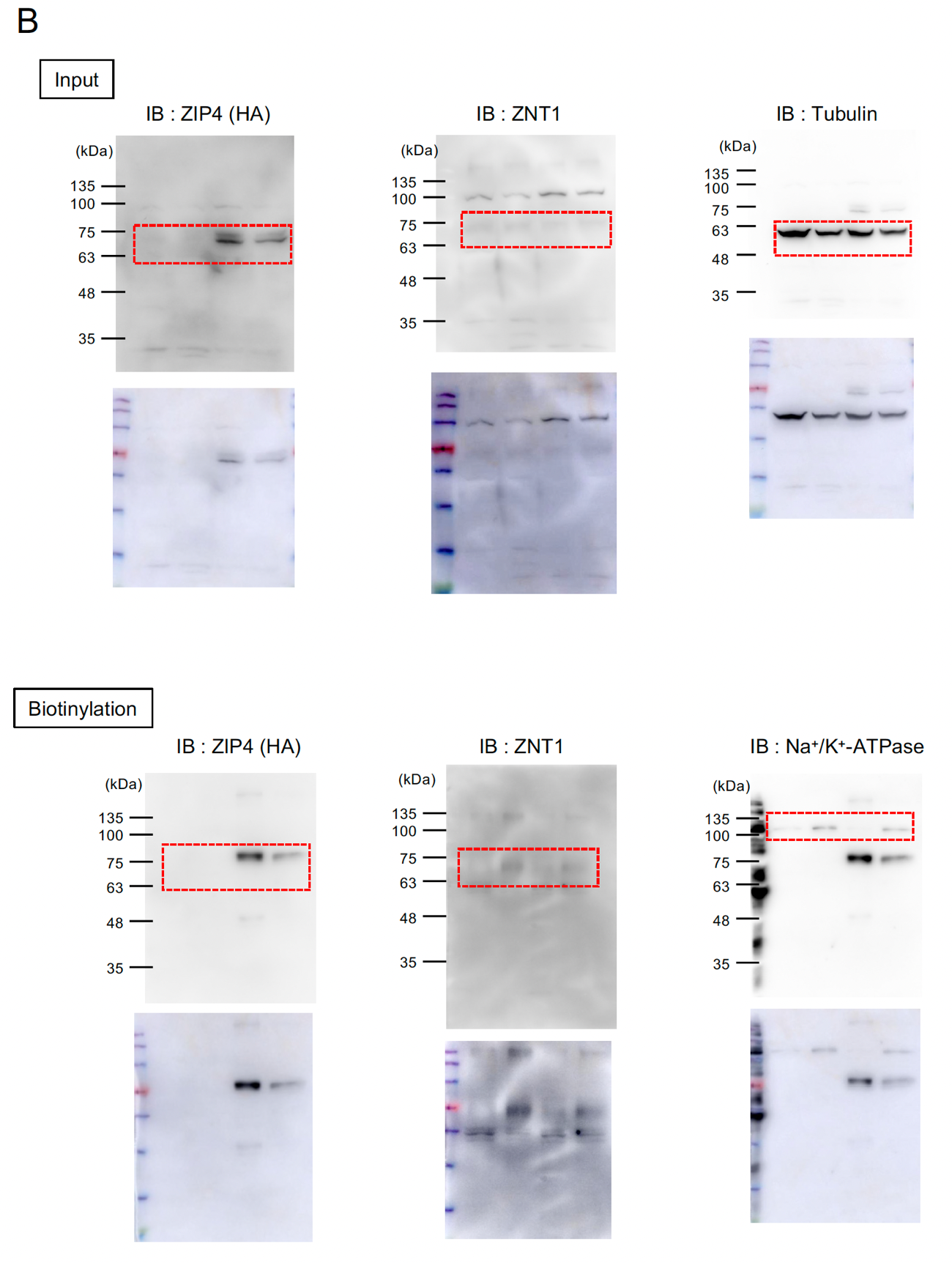
**

**Supplementary Figure 8. Full-length immunoblot images used in Figure 6.**

The panels used are boxed. The molecular weights of the marker proteins are indicated on the left of the immunoblot images. The images with varying exposures are also shown.

**
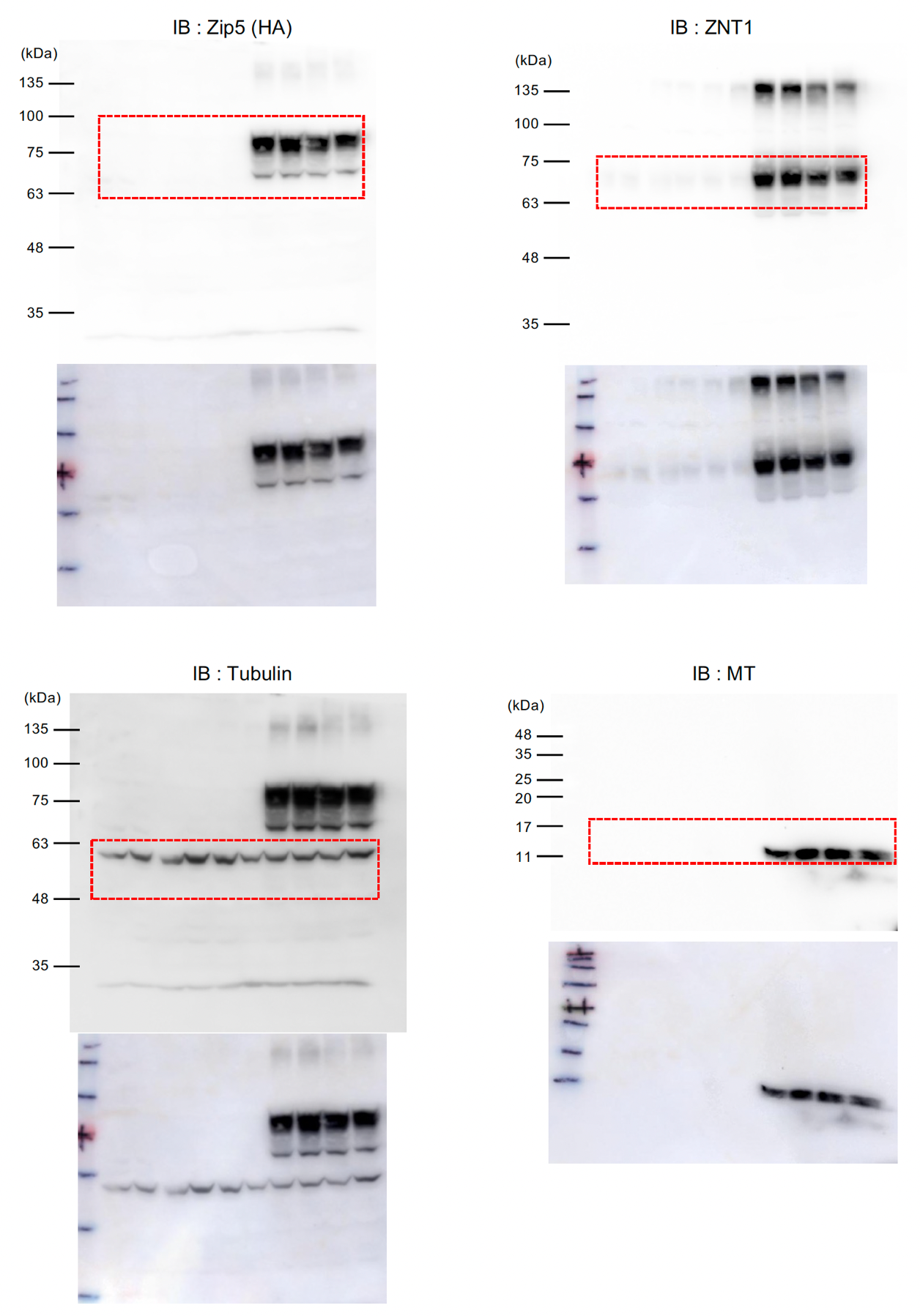
**

**Supplementary Figure 9. Full-length immunoblot images used in Supplementary Figure 1.**

The panels used are boxed. The molecular weights of the marker proteins are indicated on the left of the immunoblot images. The images with varying exposures are also shown.

**
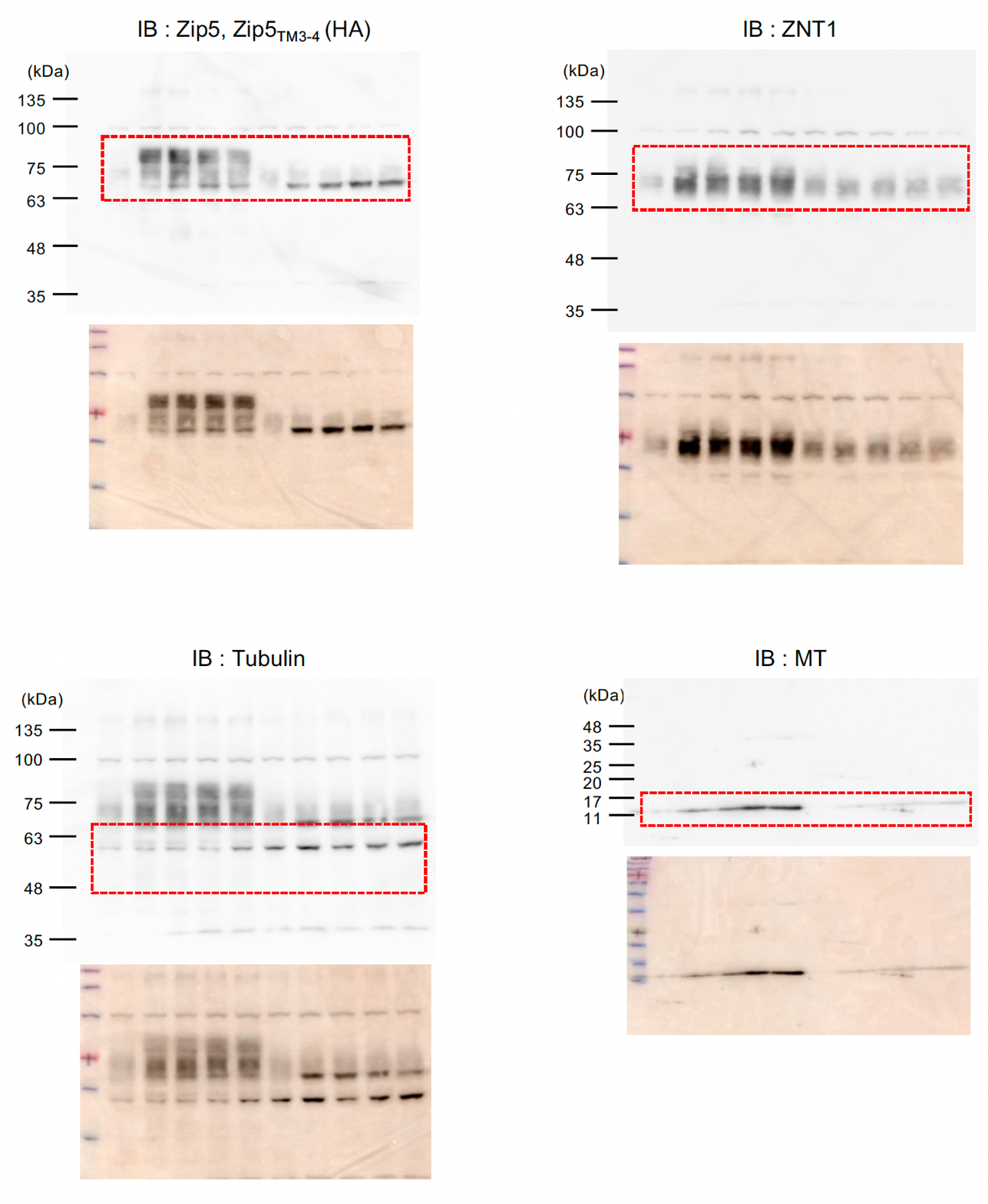
**

**Supplementary Figure 10. Full-length immunoblot images used in Supplementary Figure 2.**

The panels used are boxed. The molecular weights of the marker proteins are indicated on the left of the immunoblot images. The images with varying exposures are also shown.

**
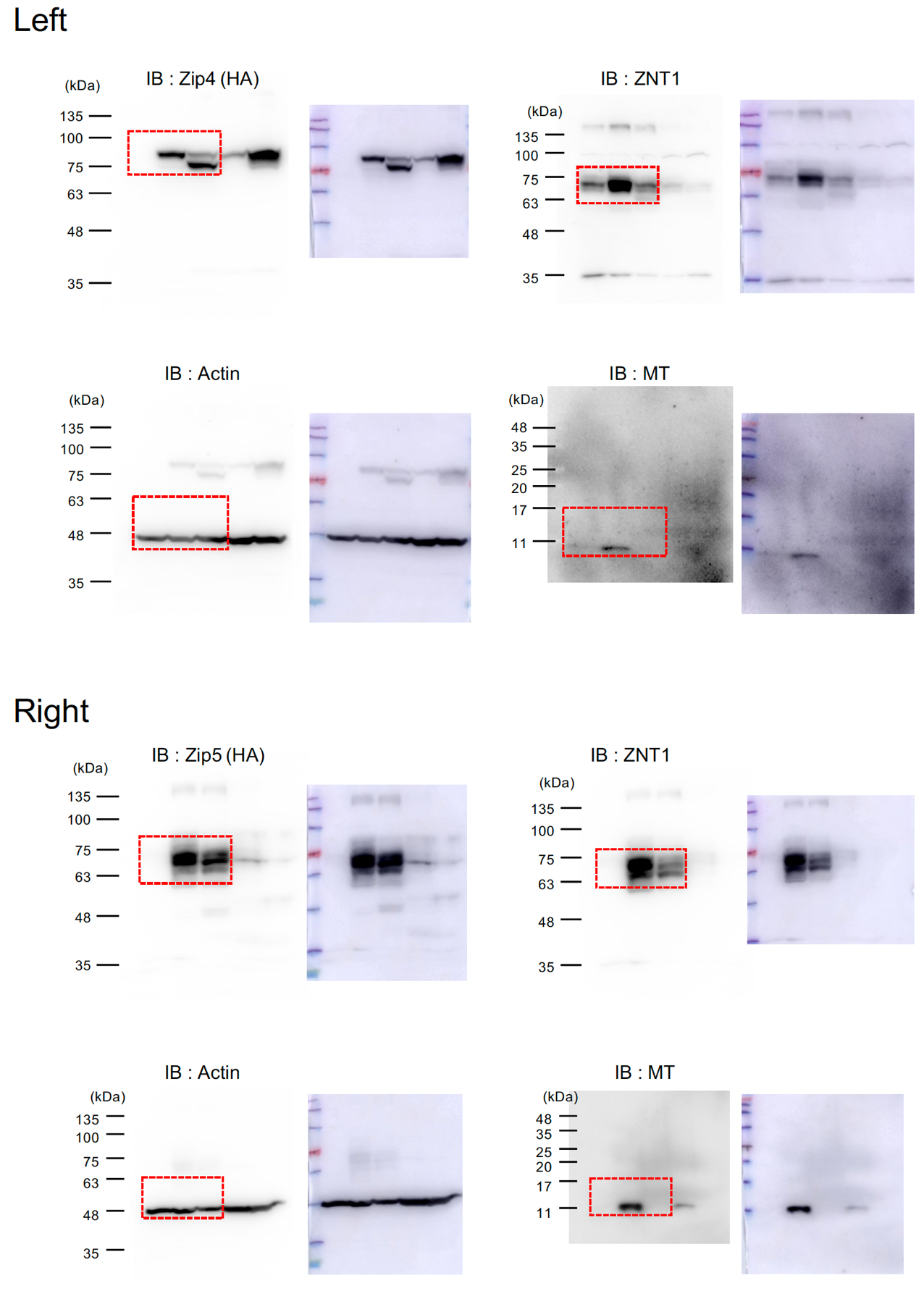
**

**Supplementary Figure 11. Full-length immunoblot images used in Supplementary Figure 3.**

The panels used are boxed. The molecular weights of the marker proteins are indicated on the left of the immunoblot images. The images with varying exposures are also shown.
